# Poly[bis­(μ_2_-*N*,*N*-di­methyl­formamide-κ^2^ *O*:*O*)bis­(μ_4_-thio­phene-2,5-di­carboxyl­ato-κ^4^ *O*:*O*′:*O*′′:*O*′′′)dicobalt(II)]

**DOI:** 10.1107/S2414314622007751

**Published:** 2022-08-12

**Authors:** Ji-Yun Ren, Rou Huang, Zheng Yin, Li-Hui Cao

**Affiliations:** a College of Chemistry and Chemical Engineering, Shaanxi University of Science and Technology, Xi’an 710021, People’s Republic of China; Dublin City University, Ireland

**Keywords:** crystal structure, metal–organic framework, cobalt(II), rod-spacer

## Abstract

The asymmetric unit of the title three-dimensional metal–organic hybrid compound, [Co_2_(C_6_H_2_O_4_S)_2_(C_3_H_7_NO)_2_]_*n*_, comprises two cobalt(II) cations, one residing on a twofold axis and the other on a centre of inversion, one thio­phene-2,5-di­carboxyl­ate (tdc^2−^) ligand and one coordinating di­methyl­formamide (DMF) solvent mol­ecule. Both of the cobalt(II) cations exhibit an octa­hedral coordination environment from the four carboxyl O atoms of the tdc^2−^ anions in a *μ*_4_-*κ* ^1^:*κ* ^1^:*κ* ^1^:*κ* ^1^ fashion and two O atoms from DMF. A pair of carboxyl O atoms and one DMF molecule connect the adjacent cobalt(II) cations into an infinite chain, leading to a rod-spacer framework with rhombus-window channels, yet no residual solvent-accessible voids are present because the coordinating DMF molecules are oriented into the potential channels.

## Abstract 2

The asymmetric unit of the title three-dimensional metal–organic hybrid compound comprises two cobalt(II) cations, one residing on a twofold axis and the other on a centre of inversion, one thio­phene-2,5-di­carboxyl­ate (tdc^2−^) ligand and one coordinated di­methyl­formamide (DMF) solvent mol­ecule. A pair of carboxyl and one DMF connect the adjacent cobalt(II) cations into an infinite chain, leading to a rod-spacer framework with rhombus-window channels, yet no residual solvent-accessible voids are present because the coordinated DMF are oriented into the potential channels.

## Graphical abstract

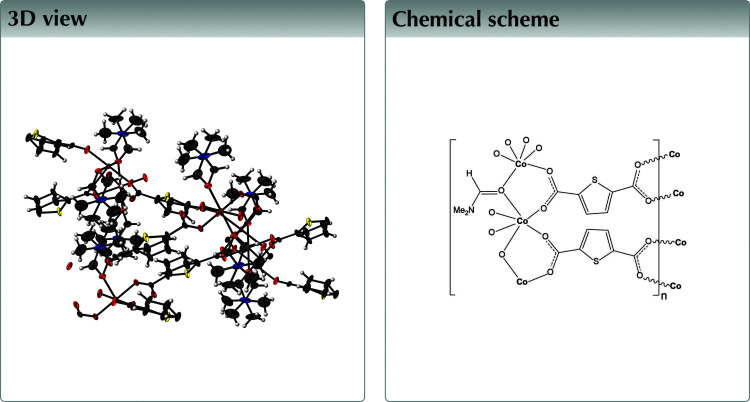

## Structure description

Facing the timetable for a carbon-neutral future, electrochemical redox reactions are the cornerstones of large-scale storage and chemical conversion of renewable clean energy in the future, in which electrocatalytic water splitting plays a central role (Seh *et al.*, 2017 ▸; Cheng *et al.*, 2022 ▸). Metal–organic frameworks (MOFs), a class of crystalline and highly porous frameworks usually constructed from 3*d* metal ions and organic ligands (Yin *et al.*, 2015 ▸), provide great opportunities for the preparation of new electrocatalysts for water splitting. Benefitting from outstanding designability and regulation for the composition and structure of MOFs, 3*d-*metal-based electrocatalysts with excellent electrocatalyst performance can be obtained from both highly stable MOFs and nanocomposites derived from the thermal or chemical reaction of the MOF precursor (Zhu *et al.*, 2018 ▸). In a previous study, we discovered alkali-induced *in situ* formation of amorphous Ni_*x*_Fe_1–*x*_(OH)_2_ from a linear [*M*_3_(COO)_6_]-based MOF template for overall electrochemical water splitting (Yin *et al.*, 2015 ▸).

In parallel work, thio­phene-2,5-di­carb­oxy­lic acid (H_2_tdc) and the cobalt ion were chosen to construct MOFs for potential electrochemical applications. The H_2_tdc ligand is a typical di-topic linker comparable to terephthalic acid that has strong coordination ability. In fact, there are 366 polymeric structure records from a total of 409 compounds constructed from H_2_tdc, based on a Cambridge Structural Database analysis (CSD version 5.4.1; December 2021; Groom *et al.*, 2016 ▸), suggesting its suitability for MOF assembly. In addition, there are carbon and sulfur elements stemming from the thia­zole ring backbone, facilitating the generation of sulfur-containing nanocomposites for electro-catalysis. On this occasion, the title compound was obtained during the synthetic exploration of new three-dimensional rod-spacer MOFs of [Co_2_(tdc)_2_(DMF)_2_]_*n*_ in a solvothermal reaction. There have been reports about the isostructural Mn^II^ compound, yet no other metal-based MOF has been described (Tan *et al.*, 2013 ▸).

**Figure 1:**
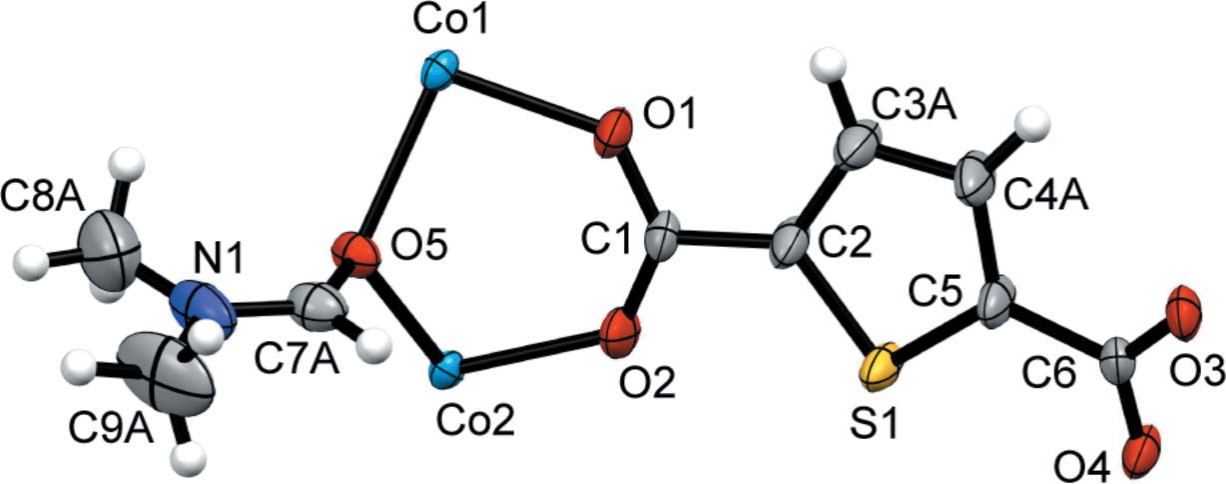
A view of the asymmetric unit of the title compound showing the atom labelling with displacement ellipsoids drawn at the 50% probability level.

**Figure 2:**
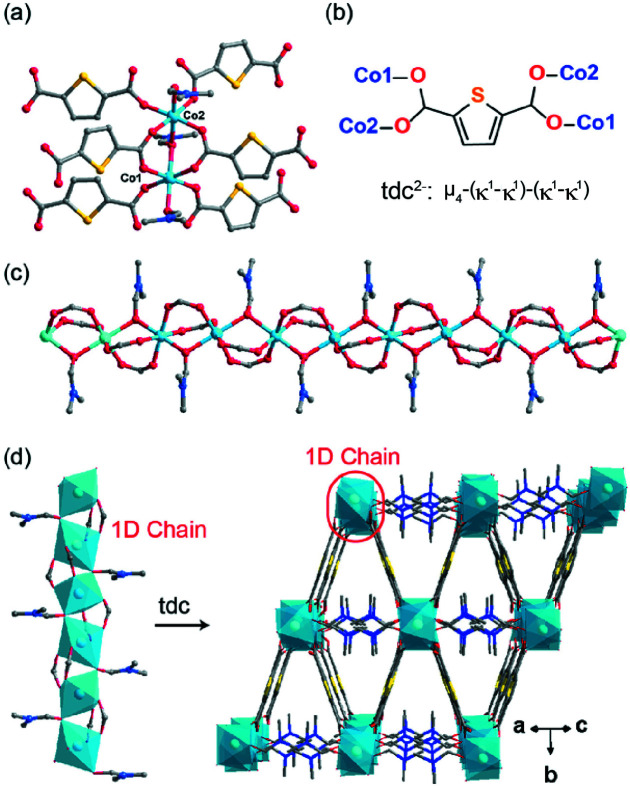
(*a*) A view of the coordination environment of the Co^2+^ ions. Colour code: Co^2+^, light blue; O, red; C, grey; S, yellow; N, blue. (*b*) The coordination modes of the tdc^2−^ ligand. (*c*) Structural view of the chain. (*d*) Perspective view of the three-dimensional rod–spacer framework.

The title compound (Fig. 1 ▸) crystallizes in the monoclinic space group *C*2/*c*. The asymmetric unit comprises two cobalt(II) cations (one resides on a twofold axis and the second on an inversion centre), one full tdc^2−^ ligand, and one coordinating DMF molecule Each of the cobalt(II) cations exhibits a octa­hedral coordination geometry by the four carboxyl O atoms from the tdc^2−^ anions in a *μ*_4_-*κ* ^1^:*κ* ^1^:*κ* ^1^:*κ* ^1^ fashion and two O atoms from DMF. The calculated continuous shape measures (CShM) value for Co1 and Co2 are 0.338 and 0.240, respectively, indicating only quite a small coordination distortion from a regular octa­hedron. A pair of carboxyl and one DMF link adjacent cobalt(II) cations into infinite chains *via* C—H⋯O hydrogen bonds (Table 1 ▸, Fig. 2 ▸). In particular, the DMF ligand adopts a *μ*_2_-bridging mode to link adjacent metal ions. Compared to its usual role as a terminally bound ligand, such coordination behaviour is rare but has been observed in some known MOFs (Fritzsche *et al.*, 2019 ▸). As a result, a rod–spacer framework with rhombus-window channels is formed through the tdc^2−^ linkage of neighbouring chains. However, no solvent-accessible voids were noted because the coordinating DMF molecule is oriented into the channels and fully occupies any potential void space. The compound is thermally stable up to 260°C under an N_2_ atmosphere by thermogravimetric analysis. Thermogravimetric analysis: the mass of the compound remains stable until 250°C, followed by an obvious mass loss of 23.7% corresponding to the loss of coordinating DMF (calculated 26.8%) in the range of 250–310°C, and then thermal decomposition of the framework with residuals of 34.3% from 400–800°C, much higher than the theoretical data for decomposition products of Co_3_O_4_ (calculated 27.7%) or CoO (calculated 26.0%), suggesting the formation of carbon- and sulfur-rich nanocomposites.

## Synthesis and crystallization

A solution of H_2_tdc (0.2 mmol, 34.4 mg) and CoCl_2_·6H_2_O (0.2 mmol, 47.6 mg) in DMF (dimethyl formamide, 15 ml) was stirred in air with a magnetic stirrer, generating a purple transparent solution after stirring for 5 min. The reaction solution was transferred to a hydro­thermal reaction vessel containing 25 ml of a polytetra­fluoro­ethyl­ene liner, followed by heating at 140°C for 48 h. The reaction vessel was cooled to room temperature at a rate of 10°C per hour. The precipitate was washed and filtered to obtain a large amount of light-purple block-shaped crystals of the title compound with a yield of about 60% (based on Co). The obtained crystals are insoluble in common organic solvents of DMF, CH_3_OH, C_2_H_5_OH, CH_2_Cl_2_ and acetone. IR (KBr pellets, cm^−1^): 3446(*bm*), 2943(*vs*), 1654(*s*), 1532(*s*), 1370(*vs*), 1106(*s*), 1010(*s*), 771(*m*), 674(*w*). Elemental analysis (%), calculated: C, 39.63; H, 3.33; N, 5.14; S, 11.76; found: C, 38.83; H, 3.76; N, 4.95; S, 12.02.

## Refinement

Crystal data, data collection and structure refinement details are summarized in Table 2 ▸. Atoms C3 and C4 of the thia­zole ring and the C atoms of the coordinating DMF are disordered over two sets of sites with occupancy ratios of 0.550 (17):0.450 (17) nd 0.855 (5):0.145 (5), respectively. Disorder treatment and restraints for the displacement parameters of the thiazole ring and coordinated DMF were applied. Disorder was treated as follows: two adjacent carbon atoms C3, C4 in the thia­zole ring were split into two parts, and the C7, C8, C9 atoms in the DMF were split into two positions also, followed by SIMU restraints for these atoms and subsequent refinements, resulting in lower, acceptable *R*-factors and refinement.

## Supplementary Material

Crystal structure: contains datablock(s) I. DOI: 10.1107/S2414314622007751/gg4009sup1.cif

Structure factors: contains datablock(s) I. DOI: 10.1107/S2414314622007751/gg4009Isup3.hkl

CCDC reference: 2178433

Additional supporting information:  crystallographic information; 3D view; checkCIF report

## Figures and Tables

**Table 1:** Hydrogen-bond geometry (Å, °)

*D*—H⋯*A*	*D*—H	H⋯*A*	*D*⋯*A*	*D*—H⋯*A*
C8*A*—H8*AB*⋯O4^i^	0.96	2.44	3.308 (6)	150
C9*A*—H9*AA*⋯O1^ii^	0.96	2.60	3.347 (6)	135
C9*B*—H9*BB*⋯O3^iii^	0.96	2.58	3.42 (4)	146

Symmetry codes: (i) 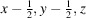 ; (ii) 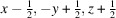 ; (iii) 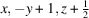 .

**Table 2:** Experimental details

Crystal data	
Chemical formula	[Co(C_6_H_2_O_4_S)(C_3_H_7_NO)]
*M*_r_	302.16
Crystal system, space group	Monoclinic, *C*2/*c*
Temperature (K)	298
*a*, *b*, *c* (Å)	11.610 (2), 18.046 (4), 11.496 (2)
β (°)	102.35 (3)
*V* (Å^3^)	2352.9 (9)
*Z*	8
Radiation type	Mo *K*α
μ (mm^−1^)	1.64
Crystal size (mm)	0.24 × 0.15 × 0.11

Data collection	
Diffractometer	Bruker APEXII CCD
Absorption correction	Multi-scan (*SADABS*; Bruker, 2016 ▸)
*T*_min_, *T*_max_	0.656, 0.746
No. of measured, independent and observed [*I* > 2σ(*I*)] reflections	12568, 2928, 2285
*R*_int_	0.054
(sin θ/λ)_max_ (Å^−1^)	0.667

Refinement	
*R*[*F* ^2^ > 2σ(*F* ^2^)], *wR*(*F* ^2^), *S*	0.040, 0.089, 1.10
No. of reflections	2928
No. of parameters	203
No. of restraints	90
H-atom treatment	H-atom parameters constrained
Δρ_max_, Δρ_min_ (e Å^−3^)	0.48, −0.44

Computer programs: *APEX2* and *SAINT* (Bruker, 2016 ▸), *SHELXT* (Sheldrick, 2015*a* ▸), *SHELXL* (Sheldrick, 2015*b* ▸), *OLEX2* (Dolomanov *et al.*, 2009 ▸) and *DIAMOND* (Brandenburg & Putz, 2019 ▸).

## full crystallographic data

### Crystal data

**Table:**

[Co(C_6_H_2_O_4_S)(C_3_H_7_NO)]	*F*(000) = 1224
*M**_r_* = 302.16	*D*_x_ = 1.706 Mg m^−^^3^
Monoclinic, *C*2/*c*	Mo *K*α radiation, λ = 0.71073 Å
*a* = 11.610 (2) Å	Cell parameters from 4391 reflections
*b* = 18.046 (4) Å	θ = 3.6–27.9°
*c* = 11.496 (2) Å	µ = 1.64 mm^−^^1^
β = 102.35 (3)°	*T* = 298 K
*V* = 2352.9 (9) Å^3^	Block, clear dark violet
*Z* = 8	0.24 × 0.15 × 0.11 mm

### Data collection

**Table:**

Bruker APEXII CCD diffractometer	2285 reflections with *I* > 2σ(*I*)
ω scans	*R*_int_ = 0.054
Absorption correction: multi-scan (SADABS; Bruker, 2016)	θ_max_ = 28.3°, θ_min_ = 2.5°
*T*_min_ = 0.656, *T*_max_ = 0.746	*h* = −15→15
12568 measured reflections	*k* = −20→24
2928 independent reflections	*l* = −13→15

### Refinement

**Table:**

Refinement on *F*^2^	Primary atom site location: difference Fourier map
Least-squares matrix: full	Hydrogen site location: inferred from neighbouring sites
*R*[*F*^2^ > 2σ(*F*^2^)] = 0.040	H-atom parameters constrained
*wR*(*F*^2^) = 0.089	*w* = 1/[σ^2^(*F*_o_^2^) + (0.0336*P*)^2^ + 4.208*P*] where *P* = (*F*_o_^2^ + 2*F*_c_^2^)/3
*S* = 1.10	(Δ/σ)_max_ < 0.001
2928 reflections	Δρ_max_ = 0.48 e Å^−^^3^
203 parameters	Δρ_min_ = −0.43 e Å^−^^3^
90 restraints	

### Special details

**Table:**

Geometry. All esds (except the esd in the dihedral angle between two l.s. planes) are estimated using the full covariance matrix. The cell esds are taken into account individually in the estimation of esds in distances, angles and torsion angles; correlations between esds in cell parameters are only used when they are defined by crystal symmetry. An approximate (isotropic) treatment of cell esds is used for estimating esds involving l.s. planes.
Refinement. The single-crystal diffraction data were collected on a on a Bruker APEX-II CCD diffractometer (Mo-Kα, λ = 0.71073?Å), with the APEX-II software for data reduction and analysis (Bruker 2016). The dataset of a selected single-crystal of (I) were collected at 298?K. The structure was solved by direct methods and refined by full-matrix least-squares method on F2 using SHELX algorithms in Olex2 (Sheldrick 2008; Dolomanov *et al.*, 2009). All non-hydrogen atoms were refined with anisotropic displacement parameters. All hydrogen atoms were generated geometrically.

### Fractional atomic coordinates and isotropic or equivalent isotropic displacement parameters (Å^2^)

**Table:**

	*x*	*y*	*z*	*U*_iso_*/*U*_eq_	Occ. (<1)
Co1	0.0000	0.22585 (3)	0.2500	0.01571 (14)	
Co2	0.2500	0.2500	0.5000	0.01521 (13)	
S1	0.28427 (7)	0.49423 (4)	0.30550 (7)	0.0294 (2)	
O1	0.11164 (18)	0.30921 (11)	0.22411 (18)	0.0282 (5)	
O2	0.24100 (19)	0.34160 (11)	0.39173 (18)	0.0292 (5)	
O3	0.21863 (18)	0.67965 (10)	0.12859 (17)	0.0238 (4)	
O4	0.38032 (18)	0.64477 (11)	0.26243 (18)	0.0266 (5)	
O5	0.06101 (17)	0.22609 (11)	0.44616 (17)	0.0226 (4)	
N1	−0.0669 (3)	0.2096 (2)	0.5674 (3)	0.0460 (8)	
C1	0.1779 (2)	0.35367 (14)	0.2909 (2)	0.0203 (6)	
C2	0.1840 (3)	0.42955 (16)	0.2402 (3)	0.0288 (7)	
C3A	0.1295 (10)	0.4528 (5)	0.1270 (9)	0.035 (2)	0.550 (17)
H3A	0.0808	0.4227	0.0719	0.043*	0.550 (17)
C4A	0.1552 (10)	0.5261 (5)	0.1043 (9)	0.035 (2)	0.550 (17)
H4A	0.1262	0.5500	0.0323	0.042*	0.550 (17)
C3B	0.1167 (12)	0.5369 (7)	0.1402 (12)	0.039 (2)	0.450 (17)
H3B	0.0643	0.5678	0.0901	0.047*	0.450 (17)
C4B	0.0916 (11)	0.4632 (7)	0.1631 (12)	0.036 (2)	0.450 (17)
H4B	0.0209	0.4396	0.1302	0.043*	0.450 (17)
C5	0.2283 (3)	0.55903 (16)	0.2002 (3)	0.0285 (7)	
C6	0.2810 (3)	0.63407 (15)	0.1970 (2)	0.0205 (6)	
C7A	−0.0030 (3)	0.2499 (2)	0.5112 (3)	0.0309 (9)	0.855 (5)
H7A	−0.0058	0.3010	0.5208	0.037*	0.855 (5)
C8A	−0.0681 (5)	0.1296 (3)	0.5558 (5)	0.0716 (17)	0.855 (5)
H8AA	−0.1193	0.1088	0.6026	0.107*	0.855 (5)
H8AB	−0.0962	0.1164	0.4737	0.107*	0.855 (5)
H8AC	0.0103	0.1107	0.5832	0.107*	0.855 (5)
C9A	−0.1409 (5)	0.2434 (4)	0.6420 (5)	0.0772 (19)	0.855 (5)
H9AA	−0.1810	0.2052	0.6758	0.116*	0.855 (5)
H9AB	−0.0922	0.2714	0.7047	0.116*	0.855 (5)
H9AC	−0.1978	0.2756	0.5941	0.116*	0.855 (5)
C7B	0.008 (2)	0.1836 (16)	0.497 (2)	0.044 (4)	0.145 (5)
H7B	0.0183	0.1328	0.4896	0.052*	0.145 (5)
C9B	−0.078 (3)	0.291 (2)	0.591 (3)	0.072 (5)	0.145 (5)
H9BA	−0.1340	0.2977	0.6413	0.107*	0.145 (5)
H9BB	−0.0030	0.3101	0.6308	0.107*	0.145 (5)
H9BC	−0.1051	0.3165	0.5175	0.107*	0.145 (5)
C8B	−0.118 (3)	0.154 (2)	0.628 (3)	0.085 (6)	0.145 (5)
H8BA	−0.1682	0.1761	0.6743	0.128*	0.145 (5)
H8BB	−0.1631	0.1202	0.5712	0.128*	0.145 (5)
H8BC	−0.0562	0.1265	0.6802	0.128*	0.145 (5)

### Atomic displacement parameters (Å^2^)

**Table:**

	*U*^11^	*U*^22^	*U*^33^	*U*^12^	*U*^13^	*U*^23^
Co1	0.0156 (3)	0.0107 (3)	0.0203 (3)	0.000	0.0029 (2)	0.000
Co2	0.0192 (3)	0.0098 (2)	0.0164 (2)	−0.0001 (2)	0.0033 (2)	−0.00102 (18)
S1	0.0331 (4)	0.0171 (4)	0.0303 (4)	−0.0084 (3)	−0.0102 (3)	0.0082 (3)
O1	0.0318 (12)	0.0204 (11)	0.0297 (11)	−0.0118 (9)	0.0004 (9)	0.0050 (8)
O2	0.0403 (13)	0.0151 (10)	0.0280 (11)	−0.0063 (9)	−0.0020 (9)	0.0069 (8)
O3	0.0254 (11)	0.0196 (10)	0.0264 (11)	−0.0019 (8)	0.0054 (8)	0.0103 (8)
O4	0.0244 (11)	0.0188 (10)	0.0335 (11)	−0.0082 (8)	−0.0009 (9)	0.0075 (8)
O5	0.0178 (10)	0.0317 (11)	0.0190 (10)	−0.0012 (8)	0.0058 (8)	−0.0002 (8)
N1	0.0297 (17)	0.083 (3)	0.0280 (15)	−0.0124 (16)	0.0124 (13)	0.0028 (15)
C1	0.0183 (14)	0.0161 (14)	0.0270 (15)	−0.0037 (11)	0.0059 (11)	0.0044 (11)
C2	0.0253 (16)	0.0224 (16)	0.0339 (17)	−0.0110 (12)	−0.0048 (13)	0.0080 (12)
C3A	0.038 (5)	0.025 (3)	0.035 (4)	−0.013 (3)	−0.012 (3)	0.008 (3)
C4A	0.040 (5)	0.027 (3)	0.032 (4)	−0.011 (3)	−0.010 (3)	0.016 (3)
C3B	0.035 (5)	0.030 (4)	0.043 (5)	−0.009 (4)	−0.013 (4)	0.018 (4)
C4B	0.030 (5)	0.029 (4)	0.042 (5)	−0.016 (4)	−0.010 (4)	0.019 (4)
C5	0.0317 (17)	0.0171 (15)	0.0309 (16)	−0.0076 (12)	−0.0062 (13)	0.0114 (11)
C6	0.0252 (16)	0.0175 (14)	0.0196 (13)	−0.0041 (11)	0.0068 (11)	0.0037 (10)
C7A	0.0246 (19)	0.042 (2)	0.0250 (18)	0.0002 (16)	0.0036 (14)	−0.0021 (15)
C8A	0.084 (4)	0.080 (4)	0.054 (3)	−0.026 (3)	0.022 (3)	0.011 (3)
C9A	0.046 (3)	0.145 (6)	0.051 (3)	0.002 (3)	0.033 (3)	−0.004 (3)
C7B	0.040 (8)	0.055 (8)	0.029 (7)	−0.009 (8)	−0.009 (7)	0.007 (7)
C9B	0.064 (10)	0.102 (10)	0.046 (9)	−0.004 (10)	0.006 (9)	−0.001 (9)
C8B	0.077 (11)	0.122 (12)	0.061 (11)	−0.021 (11)	0.027 (10)	0.004 (11)

### Geometric parameters (Å, º)

**Table:**

Co1—O1^i^	2.0485 (19)	N1—C8B	1.43 (4)
Co1—O1	2.0484 (19)	C1—C2	1.496 (4)
Co1—O4^ii^	2.0438 (19)	C2—C3A	1.385 (9)
Co1—O4^iii^	2.0437 (19)	C2—C4B	1.378 (11)
Co1—O5^i^	2.215 (2)	C3A—H3A	0.9300
Co1—O5	2.215 (2)	C3A—C4A	1.392 (11)
Co2—O2	2.0585 (19)	C4A—H4A	0.9300
Co2—O2^iv^	2.0585 (19)	C4A—C5	1.375 (9)
Co2—O3^ii^	2.0397 (18)	C3B—H3B	0.9300
Co2—O3^v^	2.0396 (18)	C3B—C4B	1.399 (13)
Co2—O5^iv^	2.192 (2)	C3B—C5	1.390 (12)
Co2—O5	2.192 (2)	C4B—H4B	0.9300
S1—C2	1.705 (3)	C5—C6	1.489 (4)
S1—C5	1.709 (3)	C7A—H7A	0.9300
O1—C1	1.254 (3)	C8A—H8AA	0.9600
O2—C1	1.251 (3)	C8A—H8AB	0.9600
O3—Co2^vi^	2.0396 (18)	C8A—H8AC	0.9600
O3—C6	1.255 (3)	C9A—H9AA	0.9600
O4—Co1^vii^	2.0438 (19)	C9A—H9AB	0.9600
O4—C6	1.250 (3)	C9A—H9AC	0.9600
O5—C7A	1.239 (4)	C7B—H7B	0.9300
O5—C7B	1.21 (3)	C9B—H9BA	0.9700
N1—C7A	1.305 (5)	C9B—H9BB	0.9600
N1—C8A	1.449 (6)	C9B—H9BC	0.9600
N1—C9A	1.469 (6)	C8B—H8BA	0.9600
N1—C7B	1.39 (3)	C8B—H8BB	0.9600
N1—C9B	1.50 (4)	C8B—H8BC	0.9700
			
O1—Co1—O1^i^	85.50 (13)	C4B—C2—S1	110.2 (5)
O1^i^—Co1—O5^i^	94.13 (8)	C4B—C2—C1	123.9 (5)
O1—Co1—O5	94.13 (8)	C2—C3A—H3A	123.5
O1—Co1—O5^i^	85.71 (8)	C2—C3A—C4A	113.1 (6)
O1^i^—Co1—O5	85.71 (8)	C4A—C3A—H3A	123.5
O4^ii^—Co1—O1^i^	175.30 (8)	C3A—C4A—H4A	123.8
O4^iii^—Co1—O1^i^	93.14 (9)	C5—C4A—C3A	112.4 (6)
O4^ii^—Co1—O1	93.14 (9)	C5—C4A—H4A	123.8
O4^iii^—Co1—O1	175.30 (8)	C4B—C3B—H3B	123.5
O4^iii^—Co1—O4^ii^	88.56 (12)	C5—C3B—H3B	123.5
O4^ii^—Co1—O5^i^	90.25 (8)	C5—C3B—C4B	112.9 (8)
O4^iii^—Co1—O5^i^	89.91 (8)	C2—C4B—C3B	112.0 (8)
O4^iii^—Co1—O5	90.25 (8)	C2—C4B—H4B	124.0
O4^ii^—Co1—O5	89.91 (8)	C3B—C4B—H4B	124.0
O5^i^—Co1—O5	179.78 (11)	C4A—C5—S1	110.4 (4)
O2^iv^—Co2—O2	180.00 (9)	C4A—C5—C6	124.1 (4)
O2^iv^—Co2—O5	86.04 (8)	C3B—C5—S1	109.1 (5)
O2—Co2—O5^iv^	86.04 (8)	C3B—C5—C6	126.4 (5)
O2^iv^—Co2—O5^iv^	93.96 (8)	C6—C5—S1	122.9 (2)
O2—Co2—O5	93.96 (8)	O3—C6—C5	115.2 (2)
O3^v^—Co2—O2	86.82 (8)	O4—C6—O3	127.6 (3)
O3^ii^—Co2—O2^iv^	86.83 (8)	O4—C6—C5	117.2 (2)
O3^ii^—Co2—O2	93.17 (8)	O5—C7A—N1	125.7 (4)
O3^v^—Co2—O2^iv^	93.18 (8)	O5—C7A—H7A	117.1
O3^v^—Co2—O3^ii^	180.0	N1—C7A—H7A	117.1
O3^ii^—Co2—O5^iv^	90.24 (8)	N1—C8A—H8AA	109.5
O3^v^—Co2—O5^iv^	89.76 (8)	N1—C8A—H8AB	109.5
O3^ii^—Co2—O5	89.76 (8)	N1—C8A—H8AC	109.5
O3^v^—Co2—O5	90.24 (8)	H8AA—C8A—H8AB	109.5
O5^iv^—Co2—O5	180.0	H8AA—C8A—H8AC	109.5
C2—S1—C5	92.08 (15)	H8AB—C8A—H8AC	109.5
C1—O1—Co1	134.86 (19)	N1—C9A—H9AA	109.5
C1—O2—Co2	130.09 (18)	N1—C9A—H9AB	109.5
C6—O3—Co2^vi^	133.92 (19)	N1—C9A—H9AC	109.5
C6—O4—Co1^vii^	128.48 (18)	H9AA—C9A—H9AB	109.5
Co2—O5—Co1	111.77 (9)	H9AA—C9A—H9AC	109.5
C7A—O5—Co1	120.9 (2)	H9AB—C9A—H9AC	109.5
C7A—O5—Co2	117.1 (2)	O5—C7B—N1	121 (2)
C7B—O5—Co1	114.2 (11)	O5—C7B—H7B	119.6
C7B—O5—Co2	124.6 (11)	N1—C7B—H7B	119.6
C7A—N1—C8A	120.3 (4)	N1—C9B—H9BA	109.5
C7A—N1—C9A	121.5 (4)	N1—C9B—H9BB	109.5
C8A—N1—C9A	118.1 (4)	N1—C9B—H9BC	109.5
C7B—N1—C9B	121.7 (18)	H9BA—C9B—H9BB	109.5
C7B—N1—C8B	115 (2)	H9BA—C9B—H9BC	109.5
C8B—N1—C9B	123 (2)	H9BB—C9B—H9BC	109.5
O1—C1—C2	114.9 (2)	N1—C8B—H8BA	109.5
O2—C1—O1	128.1 (2)	N1—C8B—H8BB	109.5
O2—C1—C2	116.9 (2)	N1—C8B—H8BC	109.5
C1—C2—S1	122.7 (2)	H8BA—C8B—H8BB	109.5
C3A—C2—S1	109.7 (4)	H8BA—C8B—H8BC	109.5
C3A—C2—C1	126.4 (4)	H8BB—C8B—H8BC	109.5
			
Co1—O1—C1—O2	38.9 (5)	C1—C2—C3A—C4A	−177.8 (5)
Co1—O1—C1—C2	−143.4 (2)	C1—C2—C4B—C3B	173.3 (6)
Co1^vii^—O4—C6—O3	−20.0 (5)	C2—S1—C5—C4A	−14.2 (6)
Co1^vii^—O4—C6—C5	160.7 (2)	C2—S1—C5—C3B	16.9 (8)
Co1—O5—C7A—N1	99.3 (4)	C2—S1—C5—C6	−176.5 (3)
Co1—O5—C7B—N1	−110.7 (16)	C2—C3A—C4A—C5	−0.6 (10)
Co2—O2—C1—O1	−8.1 (5)	C3A—C4A—C5—S1	10.9 (9)
Co2—O2—C1—C2	174.2 (2)	C3A—C4A—C5—C6	173.0 (5)
Co2^vi^—O3—C6—O4	49.8 (4)	C4A—C5—C6—O3	32.4 (8)
Co2^vi^—O3—C6—C5	−130.8 (2)	C4A—C5—C6—O4	−148.1 (7)
Co2—O5—C7A—N1	−118.5 (4)	C3B—C5—C6—O3	−3.5 (10)
Co2—O5—C7B—N1	105.9 (18)	C3B—C5—C6—O4	176.0 (9)
S1—C2—C3A—C4A	−10.0 (9)	C4B—C3B—C5—S1	−12.7 (11)
S1—C2—C4B—C3B	12.8 (10)	C4B—C3B—C5—C6	−178.7 (6)
S1—C5—C6—O3	−167.7 (2)	C5—S1—C2—C1	−177.9 (3)
S1—C5—C6—O4	11.8 (4)	C5—S1—C2—C3A	13.7 (6)
O1—C1—C2—S1	−170.0 (2)	C5—S1—C2—C4B	−17.2 (8)
O1—C1—C2—C3A	−3.6 (8)	C5—C3B—C4B—C2	0.0 (12)
O1—C1—C2—C4B	31.9 (9)	C8A—N1—C7A—O5	−0.7 (6)
O2—C1—C2—S1	8.0 (4)	C9A—N1—C7A—O5	−179.5 (4)
O2—C1—C2—C3A	174.4 (7)	C9B—N1—C7B—O5	−5 (3)
O2—C1—C2—C4B	−150.1 (9)	C8B—N1—C7B—O5	−176 (2)

Symmetry codes: (i) −*x*, *y*, −*z*+1/2; (ii) −*x*+1/2, *y*−1/2, −*z*+1/2; (iii) *x*−1/2, *y*−1/2, *z*; (iv) −*x*+1/2, −*y*+1/2, −*z*+1; (v) *x*, −*y*+1, *z*+1/2; (vi) −*x*+1/2, *y*+1/2, −*z*+1/2; (vii) *x*+1/2, *y*+1/2, *z*.

### Hydrogen-bond geometry (Å, º)

**Table:**

*D*—H···*A*	*D*—H	H···*A*	*D*···*A*	*D*—H···*A*
C8*A*—H8*AB*···O4^iii^	0.96	2.44	3.308 (6)	150
C9*A*—H9*AA*···O1^viii^	0.96	2.60	3.347 (6)	135
C9*B*—H9*BB*···O3^v^	0.96	2.58	3.42 (4)	146

Symmetry codes: (iii) *x*−1/2, *y*−1/2, *z*; (v) *x*, −*y*+1, *z*+1/2; (viii) *x*−1/2, −*y*+1/2, *z*+1/2.
